# Barriers and facilitators to insulin treatment: a phenomenological inquiry

**DOI:** 10.1186/s40545-022-00441-z

**Published:** 2022-07-19

**Authors:** Ewunetie Mekashaw Bayked, Mesfin Haile Kahissay, Birhanu Demeke Workneh

**Affiliations:** 1 Department of Pharmacy, College of Medicine and Health Sciences (CMHS), Wollo University, PO Box 1145, Dessie, Ethiopia; 2 Department of Pharmaceutics and Social Pharmacy, School of Pharmacy, College of Health Sciences, Addis Ababa University, Addis Ababa, Ethiopia

**Keywords:** Type 2 diabetes, Insulin, Barriers, Facilitators, Phenomenology, Ethiopia

## Abstract

**Background:**

Despite being the most effective treatment for advanced type 2 diabetes, the choice to start and maintain insulin therapy is based on a variety of criteria, including the patients' acceptance and willingness to adhere to it. The patients' beliefs and experiences, on the other hand, could not be revealed without a thorough exploration.

**Objectives:**

This study investigated the barriers and facilitators to insulin treatment from the perspectives of patients with type 2 diabetes following treatment at Dessie Comprehensive Specialized Hospital, North-East Ethiopia.

**Methods:**

A phenomenological study was conducted from July 2019 to January 2020. Twenty-four (11 males and 13 females) participants were recruited purposively. Data were collected through face-to-face in-depth interviews, lasted about 23 to 71 min, until theoretical saturation was reached, and then organized using QDA Miner Lite v2.0.9. The transcripts were thematically analyzed using narrative strategies and the themes that arose were discussed in detail.

**Results:**

The most common facilitator of insulin treatment was its relative effectiveness, which was followed by its convenience (fewer gastrointestinal side effects, small needle size and ease of use), the concept of it is life, faith in doctors' decisions, family support, and health insurance membership. The most common impediments, on the other hand, were market failures (expensiveness and supply shortages), followed by its properties and patients' circumstances.

**Conclusions:**

Market failures due to supply shortages and associated costs were identified to be the most significant barriers to insulin treatment, necessitating the availability of an effective pharmaceutical supply management strategy that targets on insulin supply and affordability. It is also strongly recommended that health insurance coverage be increased.

**Supplementary Information:**

The online version contains supplementary material available at 10.1186/s40545-022-00441-z.

## Background

In Africa, Ethiopia has the fourth highest number of diabetes cases (20–79 years), which grew from 1.4 million in 2011 to 1.9 million in 2021, with 1,105.9 people remaining undiagnosed. In 2021, diabetes-related expenditure per person (20–79 years) is $104.3 [1], and $17.92 per month [2]. At Dessie Comprehensive Specialized Hospital (DCSH), Ethiopia, direct medical costs accounted for 86.5% of diabetic expenditure [3].

Treatment for type 2 diabetes mellitus (T2DM) consists of lifestyle modification and nutrition therapy, exercise, and medical treatment. The mainstay medical therapies include oral antidiabetic, insulin, and non-insulin injectable medicines [4]. In Ethiopian standard treatment guideline, metformin is the first-line medical treatment for T2DM. Sulfonylureas can be used if metformin is intolerant or contraindicated. Sulfonylurea or basal insulin could be added to metformin if glycemic goals are not met after 3 months. Metformin, sulfonylurea, and basal insulin will be combined if this combination fails to control it. If it continues to be uncontrolled, multiple insulin shots per day are advised [5].

This could be because insulin production in patients with T2DM is impaired due to progressive decline or deterioration of the beta-cell function of the pancreas which eventually dictates that these patients require insulin injection to control their diabetes or to keep normoglycemia [6, 7]. Insulin therapy is therefore the best treatment option for patients with advanced T2DM [8]; that means, it is the most effective treatment for T2DM [9–11]. The East African Diabetes Study Group (EADSG) advises starting insulin therapy even in newly diagnosed T2DM patients with symptoms and/or severe hyperglycemia [12]. However, the decision to initiate and maintain insulin therapy is based on a number of issues, including the patients' beliefs and adherence [6, 13]. This is particularly challenging in countries with diverse socio-cultural backgrounds, and healthcare delivery systems [14], such as Ethiopia. For instance, the prevalence of delayed insulin initiation in patients with T2DM was estimated to be 64.2% in a study conducted at Tikur Anbessa specialized hospital due to patient, clinician, and health facility-related barriers [15]. This study therefore investigated the barriers and facilitators to insulin treatment from the perspectives of patients with T2DM following treatment at DCSH, North-East Ethiopia. The research questions were thus: What were the barriers and facilitators to insulin treatment?

## Methods

### Design and setting

A phenomenological investigation using in-depth interview data collection technique was conducted to the point of theoretical saturation (the point at which no more emerging themes were found); from July 2019 to January 2020 in Dessie City Administration (DCA) at DCSH in North-East Ethiopia. Phenomenology is a powerful approach for exploring challenging problems in healthcare [16]. It helps us in comprehending the conscious world of human beings [17]. It can be explicitly applied to the first-person experience of illness in order to illuminate the experience and enable health care workers better comprehend it [18].

Dessie is a multi-ethnic city in Northeastern Ethiopia, housing the largest and highest tertiary hospital, DCSH, with the largest catchment area. Still, it is the only referral hospital in North-East Ethiopia. It serves more than 8 million people. It includes Eastern Amhara, Afar, and Southern Tigray regions. In total, the hospital had 925 regular employees: 616 health workers and 309 administrative staff. In addition, as secondary affiliation, 15 general practitioners and 38 physicians of various specialties from Wollo University have worked. Diabetes patients are served at the diabetic clinic of the hospital [19].

### Data collection tool and procedures

A semi-structured interview guide prepared in *Amharic* (local language) was used as a tool for data collection (Table 1), using in-depth face-to-face interviews via recording with an audio device. This is because in health care research, semi-structured in-depth interviews are the most common qualitative data source [20]. The interview guide was prepared based on a comparable study carried out in Malaysia [21], but it was adjusted to fit the local contexts. It was first pre-tested with two T2DM patients using insulin treatment who were selected purposively in a different health facility, Boru Meda Hospital (BMH), and adjusted as needed. Twenty-four interviewees were selected and interviewed among T2DM patients who were following their treatment at DCSH. Participants were purposively recruited (only the T2DM patients on insulin treatment), the most popular sampling strategy used in qualitative research [22], and interviewed at their appointment time in quiet, undisturbed locations; i.e., in separate locations of the hospital campus, isolated areas of cafes, secured work areas, and participants’ homes. Participants were individuals who were chosen to represent a range of socio-demographic categories (Table 2), were mentally healthy, were not in critical conditions, did not have gestational and type 1 diabetes; who were identified by healthcare providers knowledgeable about diabetes; and willing to engage in a conversation to share their perceptions and experiences. The samples were determined by theoretical saturation, which shows the point in data collecting when no new issues or insights are found and data start to repeat so that continuing data collection is unnecessary, indicating that an appropriate sample size has been reached [23]. The time interval of the interviews ranged from 23 to 71 min with an approximate average of 46 min.

**Table 1:** Semi-structured interview guide

1.1 What do you believe diabetes is? 1.2 Do you remember what medicine was initially prescribed? 1.3 What is your experience with using insulin? 1.4 What influences did these experiences face? 1.5 How did you find injecting yourself with insulin? 1.6 How did you find the access of insulin? 1.7 Any obstacles that kept you from using insulin?

**Table 2:** Description of study participants (*n* = 24)

Description	No. of patients	Description	No. of patients
*Age (years)*		*Occupational status*	
30–39	4	Farmer	5
40–49	4	Merchant	3
50–59	6	Public servant	5
60–69	5	House wife	3
≥ 70	5	Retired	3
*Marital status*		Unemployed	5
Married	17	*Diabetes duration (years)*	
Widowed	6	1–5	6
Divorced	1	6–10	7
*Religion*		11–15	3
Orthodox	11	16–20	4
Muslim	12	21–25	2
Protestant	1	26–30	1
*Educational status*		31–35	1
Illiterate	11	*Insulin duration (years)*	
Basic literacy	4	< 1	4
Secondary school	4	1–5	13
Diploma	2	6–10	4
Graduate	2	11–15	1
Postgraduate	1	16–20	1
*Residence*		21–25	1
Urban	17		
Rural	7		

### Data processing and analysis

The interviews were transcribed verbatim into MS Word by the researchers themselves and checked again. Transcripts were read repeatedly before being translated into English to ensure good understanding. The coding and analysis procedures were started immediately after the first interview and carried out together with the data collection. The coding activity was done by repeatedly swishing back and forth to find out more emerging themes and get a detailed description of the themes.

Three analysts (EMB and other Amharic-speaking team members, MHK and BDW) delved into the data by independently reading and open-coding the transcripts and developing tentative codes. These three individuals met regularly to discuss emerging issues and refine code definitions, with regular input from each other until agreement was reached on codes and their definitions. Each transcript was coded line by line and these codes were organized into higher-order conceptual themes. Sections of the original transcripts and key quotes that were considered illustrative of the emerging themes have been translated into English to facilitate discussion between the three researchers as needed.

Memos and notes were included in the analysis to provide a more conceptual explanation. They were used to support all data analysis activities: data reduction (extracting the essence), data display (organizing by meaning), and drawing conclusions (explaining the results). Individual codes and themes were discussed at group meetings until consensus was reached. Finally, the themes were incorporated into a conceptual model of the participants and their perceptions regarding the facilitators and barriers to insulin treatment. Data analysis was thus thematically, a widely used qualitative analytic method [24], organized using QDA Miner Lite v2.0.9.

### Rigor

To ensure data trustworthiness, the transcripts were given back to three participants before being translated to English, who assured that the interpretations were their perceptions and experiences. We also complied with the “Standards for Reporting Qualitative Research (SRQR) checklist [25]” (Additional file 1).

### Reflexivity

The lead author (EMB) has been part of the community since birth and is familiar with the local slang. So, this might result in insider bias. He has been a BSc nurse practitioner and lecturer and researcher in social and administrative pharmacy for more than eight years. He therefore had a sufficient understanding of diabetes and its treatments. This could also lead to bias. Due to his literacy, the participants perceived him as a powerful individual, which could lead to outsider bias. But he used the principle of bracketing [17], and was also aware of these biases in the research process. Moreover, to minimize the biases, he used probing techniques in the in-depth interviews to delve into the participants' experiences.

## Result

### Socio-demographic description

A total of 24 participants (11 men and 13 women) were interviewed. The minimum and maximum ages of them were 35 and 75, respectively, with an approximate mean age of 55. The approximate average life spans with diabetes and insulin use of the participants were 12 and 5 years, respectively (Table 2).

### Labeling (diabetes and insulin)

Most participants were not aware of diabetes before diagnosis. In *Amharic*, diabetes mellitus is *“Ye-Siquar Beshita”,* which means *“The disease of Sugar”,* and short-term diabetes is “*Siquar”,* which means *‘Sugar’*. The term *“siquar”* is also used to express blood sugar levels as well table sugar. This is how *“siquar”* was understood contextually, whether it was meant to express diabetes or blood sugar. Each calibration point of the syringes was understood by saying *“at the number”* or just *“the number”*. This was also used to express the syringe mark where they were told to limit their dose. Insulin was simply expressed as *“an injection”*. Thus, the drug insulin and the term injection in *Amharic* were equivalently called *“Merfe”.* The syringe and needle together are called *“Mewogia”* or *“Mewugia”,* which can be interpreted as “*an apparatus to pierce the skin and deliver the drug into the body*”. *“Merfe”* is also sometimes used to express the syringe with its needle. Therefore, *"Merfe"* could be taken contextually to either express insulin or the syringe with its needle.

### Barriers and facilitators to insulin treatment

**Fig. 1:**
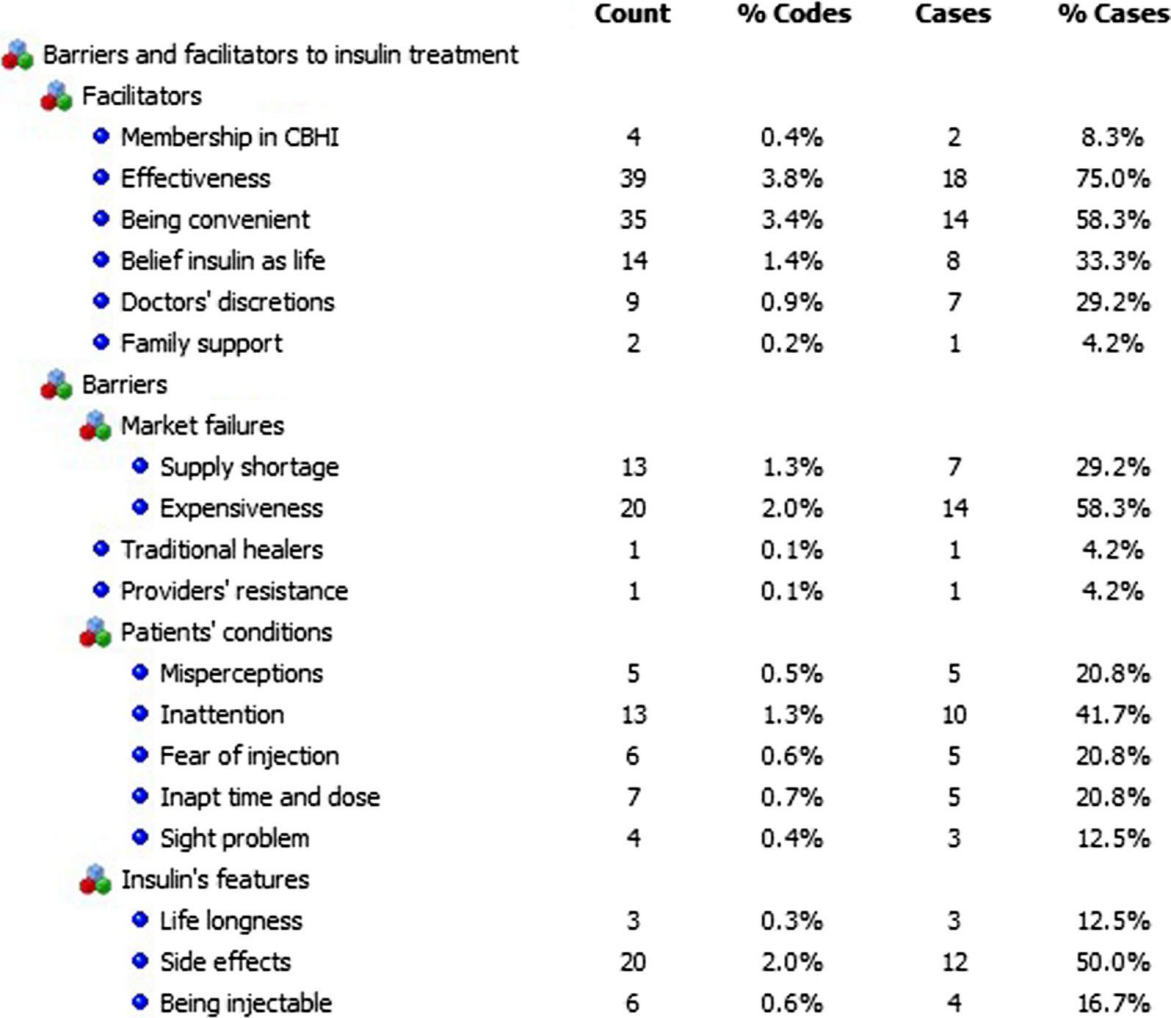
Barriers and facilitators to insulin treatment

The perceived barriers (obstacles) and facilitators (enablers) to insulin treatment were related to patients and their families, insulin and its complements, market situations, traditional healers’ interventions, payment mode or availability of health insurance and doctors’ inclinations (Fig. 1).

#### Facilitators

Insulin's relative effectiveness, convenience (fewer gastrointestinal side effects, small needles and ease of use), view of insulin as life, trust in doctors' decisions, membership in community-based health insurance (CBHI), and family support (acceptance and encouragement from family members) were all reasons that facilitated its usage. The relative effectiveness was the most frequently noted facilitator, followed by the needle's tiny size and ease of use (convenience), the impression of it as life, and respect for doctors' discretions.

Insulin, it was stated, is life, and there is nothing greater than life, thus it should be used with interest. Insulin was found to be more effective than oral hypoglycemic medications in lowering blood sugar levels. It had no strange effects, and the needles were small, light and comfortable to use. It did not have food interference, which allows the patients’ appetite to open. It was also noted that it did not cause gastrointestinal discomfort and upset as oral hypoglycemic medications did.

*It (insulin) is life; is there more than life? It is better than the oral drug. The injection (insulin) is fine and very effective to control the disease (diabetes); nothing strange with it. The needle is also light and small. (Male, 58 years, Illiterate).*

*The previous drug (oral agent) was uncomfortable to me. It made my stomach ache and distressed. It also closed my appetite and I did not eat food. But now I eat the proper diet I need. (Female, 42 years, Secondary school completed).*

Insulin, it was reported, prevents bothersome hyperglycemia symptoms such as increased thirst, frequent urination, and body discomforts (pains or aches), which improves sleep quality, which in turn improves happiness, health, and quality of life.

*I am fine. I think the “Merfe” (insulin) is my life. Because when it is compared to that time (with oral agents), I took the previous one (oral agent), my existing body is very different. Now, no increased thirst; no frequent urination; no pain. (Female, 42 years, Secondary school completed).*

*When I started insulin, many…many things have been changed. I am sleeping well; my body weight become improved. I feel pleasure and peace of mind. I thought my body demanded the treatment with insulin. (Male, 48 years, Bachelor’s degree holder).*

Insulin treatment was said to be facilitated by the doctors' discretions and the support of the patients’ families. Patients who had been persuaded by their doctors and relatives to start taking insulin were more committed to use it.

*The doctor said, no! And started me on the injection (insulin). I have just been using it afterwards. I made the decision that life was more important than anything else. It was inevitable; I convinced myself. Because it is nothing more than life. (Male, 52 Years, Secondary school completed).*

*The doctor convinced me enough to accept it (insulin) and he made me the injections carefully at first while telling me how easy it (injection technique) was and I accept it then. He (my husband) also said, “no matter what it is, if it is just a medicine for her (me); if it gives her relief; what I need is a good outcome”. Then, I have been using it with love. (Female, 38 years, Illiterate). *

The CBHI was highlighted as having helped patients who could not afford to pay for insulin out of pocket. Patients who were members stated that they would be dead without CBHI, but that because they paid for it, they could be living. They stated that they are living as long as they are members of CBHI since it protects them from costly out-of-pocket insulin costs. As a result, the participants, who were members of CBHI, have advised those patients who were not members and their neighbors to join CBHI and pay the premium right away.

*I teach health insurance (CBHI) in my Kebele. It helps; it has health benefits. Now, they (non-CBHI users) are taking my advice. I tell them that “you should hurry to pay money in advance; I live as long as health insurance works; I have health insurance and am alive unless I die. Without it, regarding money, you know I don’t have enough, but you got me in using health services. But if the payment is out of my pocket, I’m still dead”. (Male, 46 years, Illiterate).*

#### Barriers

Patients' circumstances, market failures, providers’ resistance, traditional healers, and the characteristics of insulin were all identified as barriers. However, market failures were the most typical obstacles, with cost being a major barrier secondary to supply shortage. The most frequently reported barrier to insulin treatment was its high cost, followed by insufficient supply. The hospital's supply shortage, which forces patients to buy from expensive private drug retailers, was the cause of the high price. Patients who used CBHI, on the other hand, indicated that they had no financial difficulties, except for the supply scarcity.

*Access… At the level of public hospitals, there is a significant problem. At a very low-income level, there may be a financial constraint on purchasing from a private seller. And there's a scarcity. Adapting to life is quite challenging. (Female, 42 years, Secondary school completed).*

*Still, I've been having issues with the needle for almost a year. We got it for a higher price from private pharmacies after it vanished from the hospital (DCSH). (Female, 42 years, Secondary school completed).*

It was stated that even in public healthcare facilities, many patients could not afford the cost of insulin. Patients were either obliged to ask for help from others or borrow money from them in order to purchase their medicine due to the high cost. In order to find more money to buy their medicine, they were also obliged to waste a significant amount of time.

*It is prohibitively pricey. We once encountered someone who was in a health center on his appointment date and had never been able to purchase it (insulin). He had money that was less than the fixed price. Then, we all contributed in to assist him in purchasing his medicine. (Female, 35 years, Diploma holder).*

*When I sought to purchase insulin at a government hospital (DCSH), I was told it was out of stock. Then, I went outside, but the money I had wouldn't be enough to cover the cost because it was so high. And I went home to get additional money and then came back to buy it. (Female, 35 years, Diploma holder).*

In terms of patient-related barriers, inattention was the most common concern, followed by incorrect dose and time estimation, fear of injection, misperception, and eyesight issues (problems). The frequent forgetfulness of patients to take their insulin was mentioned. It was also stated that it makes no difference whether a dose is missed or skipped. This could result in uncontrolled hyperglycemia, which in turn could have serious consequences.

*Yes, I’d forgotten. It (forgetting) does not bother me. To me, it makes no difference. What happens if I fail to take it for a single night? If it (taking insulin) is disregarded for even two days, it will have no negative effect. (Male, 65 years, Illiterate).*

*Because I assumed I'd used it, I'd always forget to use it (insulin). But I've never used it more than once. It is, by the way, forgettable. I remember that I had neglected to take it when my body bothers me. I also experience fever. Then, I asked my family to know if I utilized it or not. (Female, 35 years, Diploma holder).*

Not only was forgetting to take their medicine a concern, but so was taking repeated (double) doses, which hardly ever resulted in hypoglycemia. This could be riskier than not taking the medicine, because it could result in death if someone is not around.

*I made a mistake once. I injected it again at a later period, in the evening (double dose). Throughout the night, I was sweating profusely… I was exhausted and weakened due to severe sweating, and I was on the verge of passing out. I fell down when I attempted to get out of bed and tried to eat bread. My children were awakened by my screams and served me a sugar solution, which saved my life. (Male, 72 years, Secondary school completed).*

The potential for incorrect time and dose calculations was obvious, but the patients had no idea how the differences had developed. Patients usually utilize the dosage that was only prescribed for a month beyond that time has passed, which clearly reveals incorrect dose estimation.

*I've never finished it (insulin) before the appointment date, but I normally use it after that. However, I have never been used under dose and have no idea why it surpasses. (Female, 71 years, Illiterate).*

Another important obstacle of insulin treatment was fear of injection, with some people stating that they would rather die than be injected. Patients who were told that insulin was an injectable drug for the first time and had never heard of it before initially experienced more fear.

*Insulin was something I had never heard of before. What an exciting time that was (insulin prescription time). I was terrified of the injection (insulin); I would rather die than get injected. (Female, 38 years, illiterate).*

Misperceptions on insulin utilization with respect to injection sites were also mentioned. Erectile dysfunction (ED) was thought to be caused by injecting at the abdomen. It was believed that insulin may build up in the penis if it is administered via the abdominal route.

*It (injection site) is currently on my arm. The first one was on my abdomen. My penis died as a result of that. It (insulin) just sat there (in the body of penis) after I injected it into my abdomen. My penis had been murdered by the medication that had lingered in it for so long. Now, they (the doctors) told me to inject on my arm. Then, I switched it to my arm. (Male, 46 years, illiterate).*

Patients also thought that if they attack the disease with insulin, it will in turn revenge them in response to the harm that they imposed to it. However, they also believed that if they failed to use insulin, the disease would totally destroy them unless it has been kept to be attenuated by the medicine.

*What difference does it (insulin) make to me except it adds what I say for you? What else am I going to receive besides losing my teeth and sight? While the injection attacked it (diabetes), the disease strikes me in retaliation for the injection's (insulin’s) attack; revenges me. I, on the other hand, will be worthless if I am not injected. And the injection was beneficial. But, you know, the disease comes with loads (complications). (Male, 63 years, illiterate).*

Diabetes-related eye sight problems were noted as obstacles to insulin treatment, which imposes the assistance of others. Losing one's sight was represented as more than just a barrier to insulin use; it was also a reason for the family members' unproductivity, keeping them in charge of the patients. Therefore, for such people, having a family member nearby who is always available to help them may be a requirement in order to take their medicine.

*I won't be able to get injected unless someone is nearby. My daughter has been injecting me, and no one else has done so. No one wants to inject me if she isn't around. Despite the fact that she is well-educated, since my vision has been lost, she lives with me as if she is illiterate. (Female, 71 years, Illiterate).*

Despite the fact that doctors' discretion has been thought as a facilitator for insulin therapy, their opposition has been viewed as a hindrance. The idea is still the same, though, in that they might use their discretion as a helper and their disagreement as a hindrance for the benefit of their patients. If patients demanded insulin by themselves, doctors were supposed to be unwilling to prescribe it. Therefore, if they opposed to the initiation of insulin treatment when the clinical profile of the patient requires it, this issue may be considered as a barrier.

*The doctor didn't want me to start insulin at all. I told him the oral drug was upsetting my stomach. I wouldn't be able to eat anything. The level of "Siquar" (blood glucose) rises. Finally, I went to a private hospital and was admitted. The doctor there cautioned me that it would be hard for me to take an oral drug for the rest of my life. So, I switched to insulin. Then, I went back to the hospital (DCSH) and began to follow. (Female, 45 years, Bachelor’s degree holder).*

Traditional healers were identified as potential blockades to insulin treatment. They have led patients to stop taking insulin, believing that the treatment they have prescribed will not work until it is halted. Patients complained that some traditional healers could pressure them to quit using insulin because the healers believed that only by stopping insulin would their medication take effect. This has been cited as a major obstacle to insulin therapy and murderous behavior.

*They (traditional healers) frequently try to persuade us to abstain from taking insulin. I'm not sure why they claim insulin doesn't work when we've been using it for years. (Male, 63 years, illiterate).*

Insulin's characteristics, such as the fact that it must be taken throughout one's life, that it is injectable, and that it has adverse effects, have all been noted as hurdles to its use.

*Patients fear that it is lifelong. In fact, the tablet is also lifelong. Patients, on the other hand, frequently express their dissatisfaction with the way they live with everlasting injections. (48 Years, Male, Bachelor’s degree holder).*

*I used to say that we were about to be struck with needles for the rest of our lives. I said how terrible life is. (59 Years, Male, Master’s degree holder)*

Insulin's injectable form has been cited as a deterrent. It was mentioned that making it available in tablet form could be more convenient for patients. It has been explained why it would not be possible to create insulin in tablet form.

*It would have been preferable if insulin was available in tablet form. But, according to what I've read, it hasn't been possible yet. Even after 90 years, I read that it couldn't have been set in this way. But, why not, I say? (48 Years, Male, Bachelor’s degree holder).*

Insulin treatment faced important obstacles from its adverse effects. Its injection site pain, bleeding and burning were described as serious problems. It was also mentioned that it leaves scars.

*It is painful. It sometimes causes bleeding, and burning me. (Female, 71 years, Illiterate)*

*I've been injecting it alternately into my left and right arms. It can leave scars if used regularly. (59 Years, Male, Master’s degree holder)*

## Discussion

This study revealed that its relative effectiveness and the belief that it is life, convenience (no gastrointestinal disturbance, the needle’s small size and ease of use), and respect for doctors' discretions and family support, as well as membership in CBHI were mentioned as facilitators to insulin treatment. However, its relative effectiveness in improving quality of life (QOL) was the most frequently expressed facilitator. Similarly, according to a study conducted in Malaysia, insulin treatment was facilitated by the fact that it had fewer adverse effects, was more effective, and improved QOL [21]. Our study showed that patients' better adherence to insulin was due to its effectiveness. As also reported by a comparable study [26], their adherence was the result of its effectiveness, and their improved health was the result of their adherence. In fact, insulin is still the most effective and long-lasting hypoglycemic agent to treat advanced T2DM [27, 28].

The second most important facilitator of insulin treatment was that it is found to be convenient (fewer gastrointestinal discomfort and small needle and ease of use). Actually, if the needle is small, it is easy (convenient) to use. Other evidences also revealed that most diabetes patients preferred a smaller needle [29, 30], because it is more comfortable to use and does not need lifting a skin fold to obtain a subcutaneous injection. Small needle is found to be easy to use and enables for one-handed injections at all injection locations [30, 31]. According to the EADSG guidelines, in all patient categories, the shortest needles are the safest, most effective, and least painful option [32].

Respect for medical professionals' decisions and family support were the third critical facilitators to insulin treatment. As reported by a similar study conducted in United Kingdom [33], because doctors typically determine whether or not to initiate insulin therapy, the respect of their decisions could be important. In supporting our finding, other systematic reviews showed that family support was crucial facilitator to insulin treatment, which has been linked to better medication adherence and blood sugar control [34, 35]. On the other hand, another systematic review found that a lack of support from family and friends, as well as professionals, could have a detrimental impact on insulin treatment [36]. Hence, a systematic review [35] and a study in Peru [37] recommended that diabetes care interventions should emphasize family involvement.

Being a member of CBHI was another crucial facilitator for insulin treatment. In supporting this finding, a literature reported that diabetes patients who lack insurance are less likely to get the appropriate care; i.e., longer durations without health insurance raise the possibility of insufficient treatment for this condition and can result in uncontrolled blood sugar levels, which over time increase the risk of complications and impairment [38].

Barriers to insulin treatment were found in terms of patients’ conditions, market failures, providers’ resistance, traditional healers, and insulin's properties. According to a study conducted in Malaysia [39] and a systematic review [40], patient, healthcare professional, and system issues all had an impact on insulin treatment. The current study showed that market failures were the most common barriers, with cost being a key obstacle following a supply shortfall. Patients who used CBHI, on the other hand, said they had no financial problems other than a lack of supplies. Similarly, according to a review [41] and other original studies in Malaysia [11], Singapore [14], and India [42], cost expensiveness and financial crisis were reported to be the major barriers to insulin treatment [43]. But a study of Brazilian and Canadian patients found that they preferred that their insulin treatment be as inexpensive as possible [44]. However, according to American Diabetes Association (ADA), insulin is the most expensive diabetes treatment in terms of cash price each month, costing more than twice as much as non-insulins [45]. On the other hand, when considering the effectiveness, tolerability, and cost of other diabetic therapies, insulin is not only the most potent, but also the most cost-effective [46]. Our research found that the lack of supply of insulin and its complements (syringe with needle) were the other main market failures. In congruent to this finding, another Ethiopian study also discovered that, in government institutions, insulin frequently vanishes. As a result, patients with diabetes are forced to get it from high-cost private pharmacies [47].

In addition to market failures, the characteristics of insulin were common barriers to its usage, with the expectation of its side effects being the most frequently noted, followed by its injectability and long-term use. In supporting this finding, insulin's side effects in Oman [48], and the fact that it is a life-long therapy in Malaysia [49] and Nigeria [50] have been cited as major barriers to insulin treatment. A study in United States of America (USA) also indicated that fear of hypoglycemia was one of the most common barriers to insulin treatment [51]. So, according to a forum for injection technique held in Malaysia, all persons with diabetes who are prescribed insulin treatment should be educated about the symptoms and early management of hypoglycemia. The forum reported that lipodystrophy, particularly lipohypertrophy, is a typical issue at injection sites when insulin is given repeatedly in the same location. Therefore, as to the forum, for better glycemic control, education should also cover the importance of site rotation and evaluation of injection sites [52]. The fact that it is an injection was also highlighted, in our finding, as a deterrent to using it. Based on an evidence, in comparison to the subcutaneous route, oral administration of insulin is assumed to be the most suited and appealing [53], although many big pharmaceutical companies are still in the research and development stage [54].

The third common barriers to insulin were related to the patients’ conditions. From these, inattention was the most common barrier, followed by incorrect timing and dose estimations, fear of injection, misperceptions, and eyesight issues. Similarly, forgetting to take insulin was the leading cause of nonadherence, which is linked to worse outcomes and lower QOL [55]. According to systematic reviews [40, 56] and other findings in Singapore [14], Malaysia [11, 49], Taiwan [57], Saudi Arabia [58], and Turkey [59], fear of injection pain [11, 14, 40, 49, 57–59], fear of side effects [14, 40, 58], difficulty in self-administration, and perception of last resort [40], and misconceptions [56, 57], were all reported to be the major perceived barriers to insulin treatment. Patients in other countries have also reported that missed doses, time inaccuracy, and dose reduction are extremely prevalent. Incorrect insulin administration, such as too little, too much, or at the incorrect time, can cause transient and serious complications [60], which should be addressed during routine medical checks with appropriate advice [61]. Misperceptions about insulin use were also mentioned as barriers by studies in USA [51] and Iran [62]. The insulin to be injected in the abdomen was thought to drain and collect in the penis's body, resulting in ED. As a result, ED was misinterpreted as being caused by the buildup of injected insulin at the soma of the penis, as uniquely reported in this study. Abdominal muscle herniation, on the other hand, was rarely seen in middle-aged diabetes males [63].

Interferences from folk healers and doctors’ resistance were the least stated hurdles to insulin treatment. Similarly, according to a systematic review in Latin America [36] and an original report in Iran [62], patients' lack of faith in doctors' recommendations or inclinations for alternative remedies, on the other hand, was an impediment to modern treatment alternatives. In reality, doctors may unwittingly have a harmful impact on their patients. Putting a patient on insulin, for some doctors, indicates that he has failed to control his diabetes with oral medications and now needs to force him into a difficult treatment regime. Instead of attempting to convince their patients of the treatment's necessity, health care practitioners should analyze the precise cause for his or her patients' denial of it through active listening [51].

### Practice and policy implications

Effective pharmaceuticals management is indispensable for the availability of the right medicine at the right time and cost [64]. “Continuity of care”, a cornerstone of an effective healthcare system, is associated with improved outcomes, which require that essential medicines (including insulin) are either free or highly affordable and that tests for the diagnosing and controlling diabetes and equipment to screen for complications are available [65]. Reduction of the cost of insulin and the devices used to deliver, diagnose, and monitor it are mandatory [66]. Because access to insulin and self-monitoring are essential components of diabetes management [67], it is critical to support better planning, choose cost-effective options, and simplify the selection of priority items [66]. Misconceptions about insulin and the significance of joining CBHI must also be addressed through educational intervention, which is essential.

### Implications for future research

To develop effective strategies that could address the issue, it is crucial to consider the supply side's practical causes of market failures. Furthermore, because such a misunderstanding might frighten patients, the perception of ED as a result of the insulin to be injected in the abdomen should be studied further either to see if there is a true link or not.

### Limitations

Though in-depth exploration was used to delve into the participants’ experiences, the interview data were collected from only those who were attending their treatment at DCSH. It did not include those who are following their treatment at private hospitals. The study also did not include the health workers perceptions and experiences.

## Conclusion

Insulin use was found to be facilitated by its effectiveness, the ease with which the needle can be used, the idea that insulin is life, doctors' discretion, participation in the CBHI, and family support. Market failures (unaffordability and supply shortage) were noted as the most prevalent perceived and experienced barriers to insulin use, followed by insulin's characteristics, patients' beliefs and circumstances, involvement by folk healers, and clinicians' opposition.

## Supplementary Information

**Additional file 1.** Standards for Reporting Qualitative Research (SRQR) checklist.

## Data Availability

The data that support the findings of this study are available within the article.
